# Frequency and Predictors of Traumatic Encephalopathy Syndrome in a Prospective Cohort of Retired Professional Athletes

**DOI:** 10.3389/fneur.2021.617526

**Published:** 2021-02-23

**Authors:** Jeff Schaffert, Nyaz Didehbani, Christian LoBue, John Hart, Heidi Rossetti, Laura Lacritz, C. Munro Cullum

**Affiliations:** ^1^Department of Psychiatry, University of Texas Southwestern Medical Center, Dallas, TX, United States; ^2^Department of Physical Medicine and Rehabilitation, University of Texas Southwestern Medical Center, Dallas, TX, United States; ^3^Department of Neurosurgery, University of Texas Southwestern Medical Center, Dallas, TX, United States; ^4^Callier Center, School of Behavioral and Brain Sciences, University of Texas at Dallas, Dallas, TX, United States; ^5^Department of Neurology, University of Texas Southwestern Medical Center, Dallas, TX, United States

**Keywords:** traumatic encephalopathy syndrome, chronic traumatic encephalopathy, concussion and sports, dementia, athletes

## Abstract

Traumatic encephalopathy syndrome (TES) is proposed to represent the long-term impact of repetitive head-injury exposure and the clinical manifestation of chronic traumatic encephalopathy (CTE). This study aimed to evaluate the frequency of TES in a cohort of retired professional contact sport athletes, compare the frequency of TES to clinical consensus diagnoses, and identify predictors that increase the likelihood of TES diagnosis. Participants were 85 retired professional contact sport athletes from a prospective cohort at the University of Texas Southwestern Medical Center and the University of Texas at Dallas. Participants ranged in age from 23 to 79 (M = 55.95, SD = 13.82) and obtained 7 to 19 years of education (M = 16.08, SD = 1.03). Retirees were either non-Hispanic white (*n* = 62) or African-American (*n* = 23). Retired athletes underwent a standard clinical evaluation, which included a clinical interview, neurological exam, neuroimaging, neuropsychological testing, and consensus diagnosis of normal, mild cognitive impairment, or dementia. TES criteria were applied to all 85 athletes, and frequencies of diagnoses were compared. Fourteen predictors of TES diagnosis were evaluated using binary logistic regressions, and included demographic, neuropsychological, depression symptoms, and head-injury exposure variables. A high frequency (56%) of TES was observed among this cohort of retired athletes, but 54% of those meeting criteria for TES were diagnosed as cognitively normal via consensus diagnosis. Games played in the National Football League (OR = 0.993, *p* = 0.087), number of concussions (OR = 1.020, *p* = 0.532), number of concussions with loss of consciousness (OR = 1.141 *p* = 0.188), and years playing professionally (OR = 0.976, *p* = 0.627) were not associated with TES diagnosis. Degree of depressive symptomatology, as measured by the total score on the Beck Depression Inventory-II, was the only predictor of TES diagnosis (OR = 1.297, *p* < 0.001). Our results add to previous findings underscoring the risk for false positive diagnosis, highlight the limitations of the TES criteria in clinical and research settings, and question the relationship between TES and head-injury exposure. Future research is needed to examine depression in retired professional athletes.

## Introduction

In recent years, the long-term impact of sports-related concussion has gathered a great deal of scientific and public-health interest, in part due to the identification of chronic traumatic encephalopathy (CTE) among former National Football League (NFL) players and other professional athletes. CTE is thought to constitute a progressive neurodegenerative disease found in individuals with a history of repetitive brain trauma (1). However, there remains a great deal of controversy surrounding CTE, including a debate as to whether CTE is neurodegenerative in nature (2, 3), the risk factors for developing clinical symptoms associated with CTE (4), and the potential media bias that has accompanied recent publications (5). Furthermore, although several *in-vivo* diagnostic classifications have been proposed (6–9), CTE can only be diagnosed posthumously *via* autopsy and the clinical presentation remains poorly understood.

In 2014, Montenigro et al. proposed research diagnostic criteria for Traumatic Encephalopathy Syndrome (TES), which aimed to represent the clinical manifestation of CTE and chronic post-concussive deficits (6). These criteria were derived from a comprehensive literature review of available case series among athletes with possible CTE using Jordan's et al. criteria (8). A diagnosis of TES was “meant to describe the clinical presentation of CTE as well as other possible long-term consequences of repetitive head impacts.” The proposed diagnosis of TES consists of 5 general criteria, three core clinical features, and nine supportive features (see Table 1 for an extraction of TES criteria from Montenigro et al.). Individuals must meet all 5 general criteria, one core clinical feature, and two or more supportive features to render a diagnosis of TES. In addition, several subtypes of TES were proposed depending on the combination of the presenting symptoms, along with likelihood of CTE based on biomarker data. The three core clinical features include cognitive (attention, executive functioning, and memory) behavioral (explosive tendencies or violence), and mood symptoms (depression, apathy, worthlessness). To be considered a core clinical feature, the symptom must have been reported in at least 70% of a limited number of autopsy-confirmed CTE cases (*N* = 36) that were described in a previous publication (10). The nine supportive features include impulsivity, anxiety, apathy, paranoia, suicidality, headache, motor signs, documented decline, and delayed onset. Supportive features were selected from a literature review which identified in total 56 possible clinical features of CTE, but a more specific quantification and justification for the inclusion of these nine supportive features in the TES diagnostic criteria was not reported (6). The authors noted the selection of TES criteria was meant to favor sensitivity of the clinical features of CTE rather than specificity, and would be refined in further study.

**Table 1:** Traumatic encephalopathy syndrome clinical research criteria from Montenigro et al. (6).

**General criteria for TES**
1. History of multiple impacts to the head (or to the body resulting in impulsive force transmitted to the head). Multiple impacts are defined based upon (a) the types of injuries and (b) the source of exposure
a. Types of injuries:
i. Mild TBI or concussion, was defined according to the Zurich 2012 Consensus Statement on Concussion in Sport as a “complex pathophysiological process affecting the brain, induced by biomechanical forces…caused either by a direct blow to the head, face, neck, or elsewhere on the body with an ‘impulsive’ force transmitted to the head…the acute clinical symptoms largely reflect a functional disturbance rather than a structural injury and, as such, no abnormality is seen on standard structural neuroimaging studies. Concussion results in a graded set of clinical symptoms that may or may not involve loss of consciousness.” History of this form of trauma can be based on documented records from health-care providers or on self- or informant-reports, after being given an appropriate definition of “concussion”. If there is no reported exposure to other repetitive hits to the head, there should be a minimum of four documented mild TBIs or concussions
ii. Moderate/severe TBI was defined as having loss of consciousness of at least 30 min, alteration of consciousness/mental state of more than 24 h, post-traumatic amnesia of more than 24 h, and Glasgow Coma Scale score of <13 [59]. If there is no reported exposure to other repetitive hits to the head, there should be a minimum of two moderate/severe TBIs
iii. “Subconcussive” head trauma was defined as biomechanical forces to the head or body similar to, but less than, those required for symptomatic concussion, but without symptoms or clinical presentation consistent with concussion
b. Source of head injury exposures:
i Involvement in “high exposure” contact sports (including, but not limited to, boxing, American football, ice hockey, lacrosse, rugby, wrestling, and soccer) for a minimum of 6 years, including at least 2 years at the college level (or equivalent) or higher
ii Military service (including, but not limited to, combat exposure to blast, and other explosions as well as non-combat exposure to explosives or to combatant or breach training)
iii History of any other significant exposure to repetitive hits to the head (including, but not limited to, domestic abuse, head banging, and vocational activities such as door breaching by police)
iv For moderate/severe TBI, any activity resulting in the injury (for example, motor vehicle accident)
2) No other neurological disorder (including chronic residual symptoms from a single TBI or persistent post-concussion syndrome) that likely accounts for all clinical features, although concomitant diagnoses of substance abuse, post-traumatic stress disorder (PTSD), mood/anxiety disorders, or other neurodegenerative diseases (for example, AD and frontotemporal dementia), or a combination of these can be present
3) Clinical features must be present for a minimum of 12 months. However, if treatment (for example, “antidepressant” medication) results in an improvement in select symptoms, the clinician should use her or his best judgment to decide whether the symptoms would have persisted or progressed if treatment had not been initiated
4) At least one of the core clinical features must be present and should be considered a change from baseline functioning
5) At least two supportive features must be present
**Core features of TES**
1) *Cognitive*. Difficulties in cognition:
a) as reported by self or informant, by history of treatment, or by clinician's report of decline; and
b) substantiated by impairment on standardized mental status or neuropsychological tests of episodic memory, executive function, and/or attention, as defined by scores at a level of at least 1.5 standard deviations below appropriate norms
2) *Behavioral*. Being described as emotionally explosive (for example, having a “short fuse” or being “out of control”), physically violent, and/or verbally violent, as reported by self or informant, by history of treatment, or by clinician's report. A formal diagnosis of intermittent explosive disorder would meet this criterion but is not necessary
3) *Mood*. Feeling overly sad, depressed, and/or hopeless, as reported by self or informant, by history of treatment, or by clinician's report. A formal diagnosis of major depressive disorder or persistent depressive disorder would meet this criterion but is not necessary
**Supportive features of TES**
1) *Impulsivity*. Impaired impulse control, as demonstrated by new behaviors, such as excessive gambling, increased or unusual sexual activity, substance abuse, excessive shopping, or unusual purchases, or similar activities
2) *Anxiety*. History of anxious mood, agitation, excessive fears, or obsessive or compulsive behavior (or both), as reported by self or informant, history of treatment, or clinician's report. A formal diagnosis of anxiety disorder would meet this criterion but is not necessary
3) *Apathy*. Loss of interest in usual activities, loss of motivation and emotions, and/or reduction of voluntary, goal-directed behaviors, as reported by self or informant, history of treatment, or clinician's report
4) *Paranoia*. Delusional beliefs of suspicion, persecution, and/or unwarranted jealousy
5) *Suicidality*. History of suicidal thoughts or attempts, as reported by self or informant, history of treatment, or clinician's report
6) *Headache*. Significant and chronic headache with at least one episode per month for a minimum of 6 months
7) *Motor signs*. Dysarthria, dysgraphia, bradykinesia, tremor, rigidity, gait disturbance, falls, and/or other features of parkinsonism. If present, the modifier “with motor features” should be used
8) *Documented decline*. Progressive decline in function and/or a progression in symptoms and/or signs, based upon repeated formal testing, clinician examination, or other formal measurement (for example, informant questionnaire) for a minimum of 1 year
9) *Delayed onset*. Delayed onset of clinical features after significant head impact exposure, usually at least 2 years and in many cases several years after the period of maximal exposure. It should be noted, however, that individual cases may begin to develop the clinical features of TES during their period of head impact exposure (for example, while still actively involved in a collision sport), especially older individuals or those who have been engaged in the high-exposure activity for many years. It may also be difficult to differentiate the clinical presentation of prolonged or persistent post-concussion syndrome (pPCS) from that of TES. Therefore, there could be cases for whom there is overlap of resolving pPCS and the initial features of TES, thus masking any delayed onset of TES

The 2014 TES criteria were adapted for use in the ongoing UNITE study (Understanding Neurologic Injury and Traumatic Encephalopathy) with the aim of identifying the correlation of CTE pathology and clinical symptoms of brain donors at presumably high risk of CTE (11). A recent abstract presented at the 2020 American Academy of Neurology Conference aimed to examine the validity of TES criteria compared to the presence of CTE pathology in the UNITE study. Eleven TES symptoms were collected *via* retrospective interviews posthumously through family interview and medical record review of 302 brain donors aged 14 to 89 years old (12). TES criteria had high sensitivity, but very low specificity of 0.23 for CTE pathology, with a high percentage of those meeting criteria for TES possessing a different neurodegenerative or vascular disease. There have been no peer-reviewed clinicopathological studies validating the sensitivity and specificity of TES criteria.

The low specificity of TES criteria to CTE is not surprising for two primary reasons. First, many of the proposed symptoms of TES are common in those with and without head-injury exposure, and in patients with other diseases. For example, apathy and suicidality occur in high frequency among those with depression, and cognitive declines seen on neuropsychological testing are frequent in those with and without neurodegenerative disease. A series of studies from Iverson et al. observed high frequencies of anger control problems (13) and depression (14) among men in the general population. Apathy, disinhibition, agitation, and other behavioral control problems are also very common among those with Alzheimer's disease (15). From a cognitive perspective, one or two low scores that could be classified as “deficits” are frequently seen among even those with normal cognition when administered a comprehensive battery of neuropsychological tests (16). Second, the nature of how TES criteria were developed (i.e., case reports from retrospective next of kin interviews) likely contributes to the poor specificity of the diagnosis. Clinical diagnostic criteria for other neurodegenerative conditions, such as Alzheimer's disease, have been developed and refined over decades of prospective studies investigating clinicopathological relationships, and as such provide a much better framework for making a clinical diagnosis despite containing overlapping symptoms with other neurodegenerative diseases. TES and/or additional CTE clinical criteria currently have no validated criteria through prospective clinicopathological studies (17). Next of kin interviews are prone to multiple biases that may influence reported symptoms. For example, retrospective informant ratings of emotional symptoms (e.g., internal mood states of depression and worthlessness) are inherently speculative. Furthermore, determining the validity of cognitive changes solely based on retrospective review or informant review (i.e., without substantiated neuropsychological impairment) is extremely difficult, especially when factors such as depression and other emotional symptoms are associated with over reporting of cognitive symptoms (18). As such, the low specificity of TES criteria to CTE is likely multifactorial.

To our knowledge, the frequency of TES symptoms has not been evaluated in a prospective cohort design of those thought to be at high risk of TES and/or CTE, and there have not been any comparisons of TES diagnoses and other clinical diagnoses. Such an investigation would speak to the ease in which retired contact sport athletes may meet criteria for TES, while being diagnosed as normal *via* clinical consensus conference. Also, despite the fact that a diagnosis of TES is assumed to be associated with cortical and/or subcortical brain damage from a history of repetitive head trauma (6), there have not been any studies specifically evaluating the relationship between head-injury exposure or other characteristics and the likelihood of obtaining a diagnosis of TES.

The present study had three primary aims. First, we aimed to document the frequency of TES diagnoses and TES symptoms (see Table 1) in retired contact sport athletes using standard clinical methods (e.g., clinical interview, clinical questionnaires, neuropsychological testing, neurological exam, and neuroimaging). Second, we aimed to compare the rates of TES diagnoses to clinical consensus diagnoses of normal cognition, mild cognitive impairment, and dementia. We hypothesized, based on non-specificity of symptoms and the presence of high base rate symptoms (e.g., depression, anxiety, etc.), that we would observe a high frequency of TES diagnoses in our sample, but also find a high percentage of those diagnosed with TES determined to be “normal” *via* consensus diagnosis. Lastly, we aimed to test the hypothesis that higher levels of head-injury exposure is associated with TES diagnosis in our sample of retired contact sport athletes. For this last aim, we hypothesized that TES diagnosis would not be associated with head-injury exposure due to the non-specificity of TES symptoms to long-term head-injury exposure.

## Methods

### Participants

Participants were 85 retired professional athletes who participated in a prospective cohort study at the University of Texas Southwestern Medical Center (UTSW) and the University of Texas at Dallas (UTD) which aimed to investigate the neurocognitive impact of repetitive head-injury exposure. Athletes were recruited via local NFL player gatherings, word of mouth, and local flyers. Recruitment was open to all retired professional athletes with history of concussion and/or head-injury exposure, but were more targeted for retired NFL players given their research interest and availability. There were no specific symptomatic inclusion criteria for the study, but participants were excluded if they were found to have a history of major territory stroke, epilepsy, or pervasive developmental disorder. The study was approved by both the UTSW and UTD IRBs and all participants completed informed consent. Of the 85 athletes who participated in the study, 77 (90.6%) were retired NFL players. Of the remaining 8 athletes, 2 were retired National Hockey League (NHL) players, 3 were retired professional bull riders, 2 were retired boxers/kickboxers, and 1 was a retired professional skier. Participants ranged in age from 23 to 79 (M = 55.95, Median = 58, SD = 13.82). Age distribution of the sample was as follows: 15.3% were 40 years old or younger, 30.6% were between 41 and 55 years old, 37.6% were between 56 and 70 years old, and 16.5% were older than 70 years of age. Retirees obtained 7 to 19 years of education (M = 16.08, SD = 1.03). Retirees were mostly non-Hispanic white (*N* = 62 [72.9%]), with a sizeable minority of African-Americans (*N* = 23 [27.1%]). Head-injury exposure was defined as the number of self-reported concussions and number of concussions with loss of consciousness (LOC) as well as number of games and years played at the professional level. A concussion was defined by the clinical interviewer *via* 1997 American Academy of Neurology standards (19). Games played and years playing professionally for all retirees were obtained *via* online databases when available (obtained *via* pro-football-reference.com, pro-hockey-reference.com, or boxrec.com; see Table 2 for demographic information and athletic career info). Unfortunately, years of exposure was not initially collected in this study and is unavailable. However, online data are available from several sources due to the high-profile nature of these athletes. Additional online searches (Wikipedia, profootballarchives.com, etc.) for each athlete were used to confirm a least 6 years of contact sport participation (at least two at the college level or higher) as required by TES criteria.

**Table 2:** Demographic and career information.

	***N***	**%**	**M**	**SD**	**Min**.	**Max**.
**Age**	85	–	55.95	13.82	23	79
**Race**						
Non-Hispanic White	62	73.1	–	–	–	–
African-Americans	23	26.9	–	–	–	–
**Years education**	85	–	16.08	1.03	12	19
Employment status^*^	78					
Unemployed	9	11.5	–	–	–	–
Retired	12	15.4	–	–	–	–
Part-time	9	11.5	–	–	–	–
Full-time	48	61.5	–	–	–	–
**Career info**						
Years playing pro	85	–	8.52	4.45	1	21
Concussions	81	–	7.62	7.86	0	50
Concussions w/LOC	83	–	2.25	2.61	0	14
NFL games played	77	–	98.57	59.44	0	226

* *Percentages based on 78 retired athletes with available data; LOC, loss of consciousness*.

Participants completed a semi-structured clinical interview, neuropsychological testing, a neurological exam, neuroimaging (magnetic resonance imaging), and self-report symptom questionnaires, including measures of cognitive and psychiatric complaints. Participants underwent consensus clinical diagnosis of normal, mild cognitive impairment, or dementia by two neuropsychologists (N.D. and M.C.) and a behavioral neurologist (J.H.) utilizing the available clinical information. Standard clinical criteria were used to determine MCI diagnoses and dementia (20). Additionally, the clinical histories were reviewed and a suspected etiology was determined based on clinical features of each disorder (e.g., Alzheimer's, Lewy body disease, Frontotemporal lobar degeneration, or unknown). TES was not considered as a diagnosis during clinical consensus conferences, as our goal was to compare frequencies of clinical diagnoses to TES.

### Measures

#### General Criteria for TES

Regarding general criteria for TES (see Table 1), all participants either sustained multiple concussions or “subconcussive trauma” through at least 6 years of contact sports participation, satisfying criterion 1. We considered all participants who reported symptoms to meet criterion 2, as comorbid psychiatric and neurodegenerative disease as reported in the Montenigro criteria are acceptable comorbid diagnoses along with TES. All symptomatic participants reported clinical features longer than 12 months, which satisfied criterion 3 (6). The last two general TES criteria, criterion 4 and criterion 5, state that one core feature and two supportive features features must be present, and are discussed below.

#### Core Features of TES

TES core features were obtained via self- or informant- report, semi-structured clinical interview, neurological exam, and neuropsychological testing (see Table 3 for sources of information).

**Table 3:** Core and supportive features data collection.

**Core features**	*Cognitive*	Self or informant report
		Semi-structured clinical interview
		CVLT-II delayed free recall Z-score ≤ 1.5
		RCFT delayed free recall Z-score ≤ 1.5
		TCST Z-score ≤ 1.5
		TMTB Z-score ≤ 1.5
		WAIS-IV digit span Z-score ≤ 1.5
		CVLT-II trial 1 Z-score ≤ 1.5
	*Behavioral*	Symptom questionnaire
		Semi-structured clinical interview
	*Mood*	Symptom questionnaire
		Semi-structured clinical interview
		BDI-II ≥ 14
**Supportive features**	*Impulsivity*	Not collected
	*Anxiety*	Symptom questionnaire
		Semi-structured clinical interview
	*Apathy*	Symptom questionnaire
		BDI-II item 12 > 0
	*Paranoia*	Symptom questionnaire
		Semi-structured clinical interview
	*Suicidality*	Symptom questionnaire
		Semi-structured clinical interview
		BDI-II item 9 > 0
	*Headache*	Symptom questionnaire
		Semi-structured clinical interview
	*Motor signs*	Neurological exam
	*Documented decline*	Not collected
	*Delayed onset*	Symptom questionnaire
		Semi-structured clinical interview

Cognitive features included cognitive complaints that were collected via self-report of cognitive decline during the semi-structured clinical interview or during completion of a symptom questionnaire that collected information regarding memory, language, attention, problem solving, and daily functioning. These complaints were then examined *via* a comprehensive neuropsychological battery of tests assessing multiple cognitive domains, including episodic memory as measured by the California Verbal Learning Test-2nd Edition (21) (CVLT-II) delayed recall trial and Rey Complex Figure Test (22) (RCFT) delayed recall trial; executive functioning as measured by the Texas Card Sorting Test (23) (TCST) logical sorts and Trail Making Test B (24) (TMTB); and attention as measured by the Wechsler Adult Intelligence Scale-4th Edition Digit Span subtest (25) (WAIS-IV DS) and CVLT-II (21) Trial 1 score. Scores at least 1.5 standard deviations below the mean were classified as impaired, per TES specifications. Tests were administered and scored by a neuropsychologist (N.D.), a neuropsychology fellow/doctoral intern, or a qualified psychometrist.

Behavioral core features were collected *via* semi-structured clinical interview and self-report on the aforementioned symptom questionnaire. On this questionnaire, participants were specifically asked if they were experiencing “Emotional outbursts or uncontrollable feelings.”

Mood core features were collected *via* self-report during the semi-structured clinical interview, endorsement of “depressed mood” on the symptom questionnaire, or a Beck Depression Inventory-2nd edition (BDI-II) score of 14 or greater.

#### Supportive Features

Data were collected on seven of nine supportive features of TES. Only two supportive features were not collected or reported in this study. These included data on “impulsivity” and “documented decline” and thus were considered absent in all athletes for the purposes of this study. Documented decline was not available due to the need for repeat testing and/or evaluation at least 1 year apart, which was unavailable at baseline for our participants. Impulsivity was not collected systematically *via* our semi-structured clinical interview and/or questionnaire and thus was also not evaluated in this study. The majority of supportive features were collected *via* self-report during the semi-structured clinical interview or on the symptom questionnaire. Because severity or frequency of TES symptoms are not specified in the Montinegro et al. criteria, we did not delineate endorsement based on severity or intensity. *Anxiety* was defined as self-reported anxiety during the semi-structured clinical interview or endorsement of frequent feelings of “worry/anxiety” on the symptom questionnaire. *Apathy* was defined as difficulty with initiation on the symptom questionnaire or endorsement of item 12 on the BDI-II, which measures a loss of interest in other people or activities. *Paranoia* was collected *via* self-report during the semi-structured clinical interview or endorsement on the symptom questionnaire. A question regarding paranoia was added after the start of the study and only available in approximately half of the sample. Data on *suicidality* was collected *via* self-report during the semi-structured clinical interview or endorsement of item 9 on the BDI-II measuring suicidal thoughts or wishes. *Headache* data was obtained *via* self-report during the semi-structured clinical interview. Motor signs or parkinsonism were obtained during a neurological exam by a board-certified neurologist (J.H.). Data on the course, i.e., *delayed onset*, was collected during the semi-structured clinical interview.

### Analyses

Chi-square analyses and frequency analyses were used to compare the percentage of retired athletes who met consensus conference diagnosis for cognitively normal, MCI, or dementia and criteria for TES.

Binary logistic regressions were used to evaluate potential predictors of a TES diagnosis, which included: demographics (age, education, and race), neuropsychological scores (CVLT-II Trial 1, WAIS-IV Digit Span, TCST logical sorts, TMT B, CVLT-II delayed recall, and RCFT delayed recall), depressive symptom scores (BDI-II total score), and repetitive head-injury exposure (number of concussions, number of concussions with LOC, games played, and years played professionally). It should be noted that games played differ substantially between sports due to season length. As such, games played was restricted to only the retired NFL players. Each variable was first examined in isolation using separate binary logistic regressions, and then included in a comprehensive model to examine the relative impact of each variable while holding other variables constant. Missing data were excluded case wise for each analysis, with *p* < 0.05 set as the level of significance.

## Results

### Sample Description

Four retired athletes were missing self-reported concussion data (5%), and two retired athletes were missing data on number of concussions with LOC (3%). For concussion history and career statistics, see Table 2. Number of concussions with and without LOC, in addition to playing statistics varied widely among the retired professional athletes. Five NFL retirees had a career statistic of 0 games played, but were rostered for either 1 (*n* = 2), 2 (*n* = 2), or 3 years (*n* = 1). It should be noted that the number of concussions reported by two players were statistical outliers (i.e., 50 and 40 self-reported concussions >3.29 SD away from the mean). However, results were very similar and significance levels did not change when these outliers were removed from the analyses, and as such, results are reported with these players retained in the analyses.

Sixty-two retirees (72.9%) reported at least one cognitive complaint. Fourteen players had at least one cognitive complaint but no objective cognitive impairment. Forty-eight retirees (56.5%) had at least one score 1.5 SD below the mean on neuropsychological testing. By domain, 25 retirees (29.4%) had at least one low score on executive functioning measures, 28 (33.7%) had at least one low score on attention measures, and 28 (32.9%) had at least one low score on memory measures.

In total, 54 retirees (63.5%) met the criteria for at least one core clinical feature. Thirty-eight retirees (46.9%) met criteria definition for core clinical feature number 1 (i.e., “cognitive”), 20 retirees (23.5%) met criteria for core clinical feature number 2 (i.e., “behavioral”), and 39 retirees (45.9%) met criteria for core clinical feature number 3 (i.e., “mood”). Regarding the “cognitive” core clinical feature, which required both a cognitive complaint and a cognitive impairment, 48 players obtained a cognitively impaired score of 1.5 SD below the mean but only 38 players met the core “cognitive” clinical feature because of the absence of cognitive complaints in 10 players. Twenty-five retirees met criteria for just one core clinical feature (29.4%), while 29 (34%) met criteria for more than one. Notably, of the 39 retirees who met criteria for the “mood” core clinical feature, only 69% obtained a BDI-II score of 14 or higher (M = 18.21, SD = 10.05), with the other 31% reporting depressed mood while obtaining a BDI-II score under 13.

Regarding supplemental features, 36 (42.4%) retirees reported anxiety, 50 (58.8%) reported apathy, 7 of 37 (19%) with available data reported paranoia, 18 reported suicidal ideation (21.2%), 26 reported headaches (30.6%), and 6 were found to have parkinsonism on motor exam (7.1%). In total, 76 retirees had a mood, behavioral, and/or cognitive complaint and all symptomatic players met the delayed onset criterion (*n* = 76, 100%).

In total, 48 (56.5%) retirees met criteria for TES. If the delayed onset criteria was not considered (all of the symptomatic sample met this criteria), 38 (44.7%) retirees would still meet criteria for TES. Out of the 48 retirees who met criteria for TES, 20 (23.5%) met criteria for TES-behavioral/mood variant, 4 (4.7%) met criteria for TES-cognitive variant, 18 (21.2%) met criteria for TES-mixed variant, and 6 (7.1%) met criteria for TES-dementia. Out of 20 retirees who met criteria for TES-behavioral/mood variant, only 1 player exclusively met the “behavioral” core clinical feature and only 6 met the “behavioral” and “mood” core clinical feature, while the remaining 13 met the definition of the “mood” core clinical feature. Five retirees (5.9%) met criteria for TES with motor features specifier (see Table 4 for frequency of core and supportive features of TES in the sample). Among those 37 retirees who did not satisfy TES criteria, 9 reported no TES symptom criterion (10.5%). Thirty-one retirees (including the 9 non-symptomatic retirees) did not meet any core clinical criteria and thus did not achieve a TES diagnosis. An additional 6 retirees reported at least one core clinical feature but did not have at least two supportive clinical features, and thus did not meet TES criteria.

**Table 4:** Frequency of TES core and supportive features.

	***N***	**%**
**Cognitive complaint**	62	72.9
**Low score (<1.5 SD)**	49	56.5
Attention	28	32.9
Executive functioning	25	29.4
Memory	28	32.9
**Core features (1+)**	54	63.5
Core feature (cognitive)	38	44.7
Core feature (behavioral)	20	23.5
Core feature (mood)	39	45.9
**Supplemental feature (2+)**	63	74.1
Anxiety	36	42.4
Apathy	50	58.8
Paranoia	7	8.2
Suicidal ideation	18	21.2
Headaches	26	30.6
Motor features	6	7.0
Delayed onset	85	100
**TES diagnosis**	48	56.5
TES-Behavioral variant	20	23.5
TES-Cognitive variant	4	4.7
TES-Mixed variant	18	21.2
TES-Dementia	6	7.1
TES-Motor features	5	5.9

*TES, Traumatic Encephalopathy Syndrome*.

### Frequency of TES Diagnoses in Those Diagnosed as Normal, MCI, or Dementia

Through clinical consensus diagnosis, 63 retired athletes were diagnosed as normal (74%), 15 with MCI (18%), and 7 with dementia (8%). Of the 22 athletes with a clinical diagnosis of MCI or dementia, suspected etiologies included AD (*n* = 14), Lewy body disease (*n* = 3), Vascular (*n* = 1), and unknown etiology (*n* = 4). Retirees with and without TES diagnoses significantly differed in the frequency of consensus diagnoses of normal, MCI, or dementia (χ^2^ = 6.143, *p* = 0.046). In the 63 retired athletes diagnosed as cognitively normal through clinical consensus diagnosis, 34 (56%) met criteria for TES. In the 15 retired athletes diagnosed with MCI *via* clinical consensus conference, 8 (46%) met criteria for TES. The significant difference observed *via* chi-square analysis was driven largely by the dementia diagnoses. All 7 retired athletes diagnosed with dementia *via* clinical consensus conference met criteria for TES. To further explore this relationship, dementia and MCI categories were collapsed into a “cognitively impaired” diagnosis vs. normal. No significant differences were found in the ratios of cognitive impairment *via* consensus diagnosis compared to those with and without TES (χ^2^ = 0.620, *p* = 0.431). Using this collapsed category, 22 retired athletes were diagnosed as having MCI or dementia through clinical consensus conference, 14 (64%) of which met criteria for TES (see Table 5 for chi-square results and frequency analyses).

**Table 5:** Chi-square analyses of TES diagnosis and consensus diagnosis.

	**Normal (*n* = 63)**	**MCI (*n* = 15)**	**Dementia (*n* = 7)**	**Chi-square**	***p*-value**
TES–	29 (46%)	8 (53%)	0 (0%)	6.143	0.046^*^
TES+	34 (54%)	7 (47%)	7 (100%)		
	**Normal (*****n*** **=** **63)**	** impaired (*****n*** **=** **22)**		**Chi-square**	***p*****-value**
TES–	29 (46%)	8 (36%)		0.620	0.431
TES+	34 (54%)	14 (64%)			

* *p < 0.05*.

To evaluate if subtypes of TES differed by age, a chi-square of TES subtypes by age (median split of ≤58 and >58 years old) was conducted (see Table 6). No statistically significant differences in frequencies were observed between subtypes of TES and a median split of age (χ^2^ = 1.698, *p* = 0.791).

**Table 6:** Frequencies of TES stratified by age.

		**TES variant**					**Total**
		**No TES**	**TES-BMv**	**TES-COGv**	**TES-MIXv**	**TES-D**	
Age	<58	17	12	2	9	2	42
		45.9%	60.0%	50.0%	50.0%	33.3%	49.4%
	≥58	20	8	2	9	4	43
		54.1%	40.0%	50.0%	50.0%	66.7%	50.6%

### Demographic, Cognitive, Mood, and Head-Injury Exposure Predictors of TES Diagnoses

Binary logistic regressions were used to evaluate each of the 14 predictors (Age, race, education, number of concussions, number of concussions with LOC, years playing professionally, games played, CVLT-II Trial 1, WAIS-IV Digit Span, TCST logical sorts, TMT B, CVLT-II delayed recall, RCFT delayed recall, BDI-II total score) independently prior to including it into a comprehensive model. Worse performance on CVLT-II trial 1 was associated with TES diagnosis (B = −0.576, Wald χ^2^ = 5.754, OR = 0.562 [0.351–0.900], *p* = 0.016) in addition to higher endorsement of depression symptoms on the BDI-II (B = 0.260, Wald χ^2^ = 17.432, OR = 1.297 [1.148–1.465], *p* < 0.001). The remaining univariate binary logistic regressions were not statistically significant (all *p*'s > 0.05—see Table 7 for binary logistic regressions).

**Table 7:** Univariate binary logistic regressions assessing demographic, head-injury exposure, cognitive, and mood predictors of TES diagnosis.

	***N***	**B**	**SE**	**Wald**	**Exp(B)**	**95% CI**	***p***
**Demographic**							
Age	85	−0.016	0.016	0.958	0.984	0.954–1.016	0.328
Race	85	0.499	0.506	0.972	0.607	0.225–1.637	0.324
Education	85	0.002	0.214	0.000	1.002	0.658–1.526	0.992
**Head-injury exposure**							
No. of concussions	81	0.019	0.031	0.391	1.020	0.959–1.082	0.532
No. of concussions w/LOC	83	0.132	0.100	1.731	1.141	0.938–1.388	0.188
Years played professionally	85	−0.024	0.050	0.236	0.976	0.886–1.076	0.627
Games played (NFL only)	78	−0.007	0.004	2.922	0.993	0.985–1.001	0.087
**Neuropsych performance**							
WAIS-IV digit span (Z)	85	−0.062	0.214	0.083	0.940	0.618–1.431	0.774
CVLT-II trial 1 (Z)	83	−0.576	0.240	5.754	0.562	0.351–0.900	0.016^*^
TCST sorts (Z)	78	−0.206	0.201	1.049	0.814	0.549–1.207	0.306
TMTB (Z)	85	−0.261	0.188	1.911	0.771	0.533–1.115	0.167
CVLT-II delay recall (Z)	83	−0.198	0.182	1.180	0.820	0.574–1.173	0.277
RCFT delay recall (Z)	84	−0.119	0.139	0.734	0.888	0.677–1.165	0.392
**Depressive symptoms**							
BDI-II	83	0.260	0.062	17.432	1.297	1.148–1.465	<0.001^**^

*WAIS-IV, Wechsler Adult Intelligence Scale −4th Edition; CVLT-II, California Verbal Learning Test−2nd edition; TCST, Texas Card Sorting Test; TMT B, Trail Making Test Part B; RCFT, Rey Complex Figure Test; BDI-II, Beck Depression Inventory−2nd edition*.

* p < 0.05;

** *p < 0.001*.

When evaluating the 13 predictors (games played was dropped due to extreme differences in season length between sports) in a multivariate binary logistic regression model, the overall model was significant (χ^2^ = 46.295, *p* < 0.001) with good fit (Hosmer and Lemeshow Test *p* = 0.932; Nagelkerke R^2^ = 0.628). The classification accuracy improved from 59.5% in the null model to 84.9%. However, only BDI-II score significantly predicted TES diagnoses (B = 0.379, Wald χ^2^ = 12.107, OR = 1.461 [1.180–1.809], *p* < 0.001). No demographic, head-injury exposure variables, or neuropsychological scores significantly predicted TES diagnosis (all *p*'s < 0.05). To further examine if any additional variables improved specification of the model, the model was evaluated in a forward step-wise fashion with *p* < 0.05 as the entry criteria, and only BDI-II scores was statistically significant (B = 0.250, Wald χ^2^ = 15.542, OR = 1.284 [1.134–1.454], *p* < 0.001) out of the 13 predictors (see Table 8 for additional statistics on the multivariate binary logistic regression).

**Table 8:** Multivariate binary logistic regression assessing demographic, head-injury exposure, cognitive, and mood predictors of TES diagnosis (*N* = 74).

**Predictors**	**B**	**SE**	**Wald**	**Exp(B)**	**95% CI**	***p***
Age	−0.010	0.033	0.095	0.990	0.927–1.57	0.758
Race	0.426	0.974	0.191	1.530	0.227–10.312	0.662
Education	0.790	0.464	2.899	2.204	0.887–5.475	0.089
No. of concussions	0.027	0.076	0.131	1.028	0.886–1.193	0.718
No. of concussions w/LOC	−0.095	0.196	0.237	0.909	0.620–1.334	0.626
Years played professionally	0.229	0.133	2.945	1.257	0.968–1.632	0.086
WAIS–IV digit span (Z)	0.412	0.464	0.790	1.510	0.609–3.747	0.374
CVLT–II Trial 1 (Z)	−0.055	0.367	0.023	0.946	0.461–1.941	0.880
TCST sorts (Z)	0.403	0.446	0.815	1.496	0.624–3.587	0.367
TMTB (Z)	0.029	0.396	0.006	1.030	0.474–2.237	0.941
CVLT–II delay recall (Z)	−0.623	0.497	1.571	0.536	0.202–1.421	0.210
RCFT delay recall (Z)	−0.305	0.279	1.201	0.737	0.427–1.272	0.273
BDI-II	0.379	0.109	12.107	1.461	1.180–1.809	<0.001^**^

*WAIS-IV, Wechsler Adult Intelligence Scale−4th Edition; CVLT-II, California Verbal Learning Test−2nd edition; TCST, Texas Card Sorting Test; TMT B, Trail Making Test Part B; RCFT, Rey Complex Figure Test; BDI-II, Beck Depression Inventory−2nd edition*.

** *p < 0.001*.

An additional *post-hoc* analysis was conducted to analyze BDI-II scores and likelihood of obtaining a TES diagnosis. BDI-II scores were removed from the TES diagnostic criterion for the purposes of this analysis in order to assess if this association would remain significant if BDI-II scores were not considered in the diagnosis of TES. By excluding BDI-II in the criterion, TES frequency reduced from 48 out of 85 (56.8%) to 43 out of 85 (50.5%). BDI-II score remained a significant predictor of TES diagnosis, B = 0.089, Wald χ^2^ = 9.718, OR = 1.094 (1.034–1.157), *p* = 0.002) and increased classification accuracy from 51.8 to 67.5%.

## Discussion

This study aimed to evaluate the frequency of TES diagnoses and TES symptoms collected prospectively in a cohort of retired professional athletes with head-injury exposure, compared the frequency of TES diagnoses to clinical consensus diagnoses, and evaluated predictors of TES diagnosis. To our knowledge, this was the first study to evaluate the frequency of TES diagnoses and TES criterion obtained through direct clinical evaluations of living retired professional athletes with head-injury exposure. We found a high percentage of the retired athletes in our sample met criteria for TES (56%), and over half of the athletes (54%) who met criteria for TES were diagnosed as normal based on comprehensive clinical evaluation and interdisciplinary consensus diagnosis. Furthermore, the only significant predictor of TES diagnosis was level of depressive symptomatology. No significant associations were found between likelihood of TES diagnosis and demographic characteristics, head-injury exposure, or neuropsychological functioning. Our findings underscore the limitations of the TES criteria, and call into question the utility of using these criteria in clinical and research settings, due to the potential for false positive determinations.

Although there have been several investigations linking head-injury exposure and CTE pathology (26, 27), to our knowledge there have been no investigations of head-injury exposure and likelihood of obtaining a diagnosis of TES. Because TES is considered to reflect long-term difficulties related to repetitive head-injury, it is logical to assume that increased head-injury exposure would increase likelihood of a TES diagnosis. However, this study did not observe a relationship between TES diagnoses and years playing professional sports, games played in the NFL, number of concussions, or number of concussions with LOC. We also did not find an association between neuropsychological functioning and likelihood of TES diagnosis in our multivariate model. Research investigating the link between head-injury exposure and cognitive functioning has been equivocal at best (28). A well-cited study by Stamm et al. in 2015 suggested worse executive functioning deficits in those who began playing football earlier than 12 years of age (29), though this study has limitations in the form of inequity between groups in regards to learning disabilities, substance use, and steroid use, as well as the lack of a control group (30). A 2016 study by Solomon et al. and a 2019 study by Fields et al. failed to find a statistically significant linear association between head-injury exposure and neuropsychological functioning among retired NFL players (30, 31). Our findings did, however, suggest that greater depressive symptomatology increased the likelihood of TES diagnosis in our sample, which appear to be heavily emphasized in TES diagnoses (e.g., “mood variant,” apathy, worthlessness, anxiety, etc.) and are not surprising in this context.

Large scale survey studies have suggested that more concussions are associated with a higher prevalence of self-reported depression in retired NFL players (32, 33). Despite this, investigations utilizing objective psychometric measures to assess the relationship between depression and head-injury exposure have been more mixed. A 2013 study found that number of concussions was specifically associated with cognitive symptoms of depression (e.g., pessimism, feelings of guilt, self-dislike) in retired NFL players (34), and a follow-up investigation found depression symptoms to be significantly associated with white matter changes (35). However, other investigations have failed to find an association between depressive symptoms and number of concussions or years played in the NFL (31), or between depressive symptoms and years of pre-high school football (30). In a recent article by Brett and colleagues, 43 retired NFL players aged 60 or younger were administered the BDI-II (M = 9.26) and the PHQ-15 to measure somatic symptoms. The authors found that somatic symptom reporting significantly moderated the relationship between concussions and depressive symptoms, such that somatic symptoms had an ~2-fold influence on depressive symptom reporting compared to concussion history (36). Their findings suggest that additional factors beyond head-injury exposure may be responsible for the relationship between head-injury exposure and depressive symptoms, and underscore the importance of future investigations aimed at examining depression and moderators of depression following head-injury exposure in contact sport athletes.

The multifactorial etiology of depressive symptoms and the presence of depression in other neurodegenerative diseases (e.g., AD, Parkinson's disease, etc.) adds additional concerns regarding the specificity of depression for its inclusion as a core diagnostic criterion for TES. Development of clinical diagnostic criteria for a neurodegenerative disorder that may share features with other diseases requires clear consensus on the most pertinent clinical features of the disorder. Ideally, this is informed through thorough study of many patients with the suspected disorder and longitudinal follow up of individuals in whom pathological confirmation can be made, so that clinical-pathological correlates can be reviewed to help refine the diagnostic process. Brett and colleagues (18) compare the processes in which clinical and neuropathological diagnoses of CTE have been derived in comparison with disorders such as AD and Lewy body disease (LBD), highlighting the rigorous approach needed to develop criteria that have good sensitivity and specificity and are clinically useful. For example, clinical and neuropathological criteria for AD and LBD have undergone numerous iterations (AD = 8; LBD = 4), involving diverse consensus work groups spanning many different institutions and followed by validation studies. While the clinical criteria for AD have remained largely similar over time, yet better refined, the pathological criteria have clearly evolved through this process. LBD is an example of a neurodegenerative disorder in which initial clinical criteria were not very specific, resulting in many inaccurate diagnoses. Through refinement of core and supportive clinical features over time (e.g., moving rapid eye movement behavioral disorder from a supportive to core feature), the likelihood of making an accurate diagnosis of LBD has improved considerably (accuracy of diagnosis 82 to 91%). CTE is in its infancy with respect to development of precise diagnostic criteria, which has to date relied heavily on a small number of cases reviewed by a limited number of professionals for the pathological diagnosis and largely non-empirical methods of deriving clinical syndromic criteria. Exclusion of other potential causes for the various presenting symptoms is part of the diagnostic criteria for AD and LBD, underscoring that other disorders can share similar features and need to be ruled out. While the criteria for TES also includes the exclusion of other disorders that can account for all of the clinical features, allowing comorbid disorders such as AD and other dementias, as well as various psychiatric disorders almost guarantees a high overlap in symptoms when these disorders are present.

In the current TES diagnostic framework, general criterion 2 states “No other neurological disorder (including chronic residual symptoms from a single TBI or persistent post-concussion syndrome) that likely accounts for all clinical features, although concomitant diagnoses of substance abuse, post-traumatic stress disorder (PTSD), mood/anxiety disorders, or other neurodegenerative diseases (for example, AD and frontotemporal dementia) or a combination of these can be present.” When this criterion was applied to the current sample, it became difficult to discern if the presence of other neurological disease may or may not be exclusionary criteria on a case by case basis, especially in the presence of potential neurodegenerative disease and lack of specificity of the core and supportive features criteria. As written, if a clinician suspects that even a single supportive feature may not be due to a comorbid neurodegenerative disease (such as AD), this would satisfy this criterion. Almost all of the supportive features for TES (e.g., impulsivity, apathy, anxiety, paranoia, and motor signs) may or may not be present in neurodegenerative conditions such as AD (15), making it difficult to determine if AD “accounts for all clinical features.” The Montenigro et al. criteria outline two case examples that meet criteria for AD as well as TES, both of which were diagnosed with TES-dementia subtype, which suggest that comorbid neurodegenerative conditions are often present. In addition, this criterion as written is not clear if commonly referred to psychiatric conditions such as post-traumatic stress disorder, major depressive disorder, or generalized anxiety disorder are considered “neurological diseases” given their mention in the same sentence, and can be particularly difficult to distinguish as TES criteria are heavily influenced by psychiatric symptoms. If these criteria are applied as written, any retired professional contact sport athlete who had onset of depressed mood 2 years following the end of their career would easily meet criteria for TES. The ease with which retired athletes can meet criteira for TES, which was designed to represent clinical features of the neurodegenerative disease CTE, is not suprising when poor specificity, allowance of comorbid conditions that may account for the majority of clinical features, and the high base rates of many of the core and supportive features are considered.

Several of the proposed clinical features for TES are non-specific and relatively common conditions/traits occurring in the general population. For example, among 3,933 males in a national survey on mental disorders, 15% reported having an alcohol use disorder, 16% a depression-related mood disorder, 13% an anxiety disorder, 25% aggression/hostility, and 10% anger during their lifetime (13). In the normative database of the National Institutes of Health Toolbox, anger (25%) and some aggression/hostility (11%) within the past month were also common for the male cohort (37). Thus, many of the psychological problems that have been proposed for TES are not unique, and there have been no studies to date comparing even active professional athletes to the general population on base rates of the purported conditions/traits. It is possible that professional athletes may be prone to having some of the conditions/traits at higher rates than the general population, which has been found for alcohol/substance use disorders (38). For contact sport athletes specifically, it is reasonable to think that aggression/hostility could also be more prevalent, especially since there is typically a lifetime of playing fairly aggressive sports. With that said, retired professional athletes could be more prone to developing these features during aging, but the underlying etiology may not necessarily be a neurodegenerative disease. For 101 and 206 males meeting diagnostic criteria for Major Depressive Disorder and Intermittent Explosive Disorder in a larger psychological disorders national survey, ~35% met core and supplemental features criteria for TES (13, 14). This was due to irritability (48%), aggression/hostility (31%), anxiety (56%), alcohol use disorder (25%) and suicidal ideation (10%) in the past 12 months, with apathy (18%) and headaches (22%) frequently co-occurring in individuals with such psychological problems. Therefore, many of the clinical features for TES are co-morbid with psychological problems, which would make it challenging to have good sensitivity/specificity in classifying TES vs. unrelated psychological conditions. Indeed, retired professional athletes may be susceptible to experiencing problems adjusting to a shortened career, chronic physical problems, and other life stressors during aging. Misattributing symptoms to a neurodegenerative disorder rather than a treatable condition can have serious consequences. Moving forward, it will be important for future research with TES criteria to identify clinical features that are distinct from common psychological conditions/traits in the general population.

The extraordinary media coverage conflating sports, concussion, and CTE has colored both public and clinician perception of this issue, and influenced decision-making (e.g., avoiding sports, seeking financial restitution). In this context, it is possible that more common disorders or diseases may be overlooked or dismissed in the differential diagnosis, and symptoms could be instead attributed to a poorly understood, rare neuropathological entity, in stark contrast to basic diagnostic approaches across disciplines and despite repeated calls for caution (39). Common comorbidities that overlap with TES, including depression, anxiety, and substance abuse, have an array of effective, empirically based treatments and a diverse range of causes, and it is possible that a diagnosis of TES may influence either course of treatment and/or motivation to participate in treatment. Similarly, some individuals may misinterpret symptoms such as headaches, anger, and apathy as signs of an untreatable progressive disease related to past head impact exposure and fail to present clinically for proper evaluation and intervention. Furthermore, retired players have high rates of depression, chronic pain, and opioid use, and those with this constellation of problems also report greater financial strain and life stress, and it is not difficult to imagine that adding a premature diagnosis based on broad and non-specific criteria to this mix may have adverse or iatrogenic effects [for a review of suicide and CTE, see Iverson 2016—(40)]. This has been tragically illustrated by cases in which athletes have died by suicide after being told they had CTE or after their personal online research led them to this conclusion (41). Thus, determining the specific cause of these commonly occurring symptoms in any one individual can be difficult, and practitioners should be wary of providing false-positive diagnoses of progressive, neurodegenerative diseases.

Although the present study has numerous strengths, including prospective collection of potential TES symptoms, a wide age-range of retirees, and the richness of the available clinical data, there a number of limitations. First, as is the case with most studies, concussion data were collected *via* self-report and was not verifiable and may be prone to recall bias. Second, the current study did not collect data on impulsivity, although this likely would have increased the already high frequency of TES diagnoses in the sample. Third, because the sample was recruited without strict criteria, it is possible that word of mouth among the retirees participating may have increased the recruitment of other retirees with neurological and psychiatric disorders due to the clinical nature of the study. As such, although we cannot speak to the prevalence of TES diagnoses in all retired professional contact sport athletes, our findings still underscore the ease at which normally functioning retired athletes can be diagnosed with a syndrome purported to represent a neurodegenerative disease. Fourth, in the logistic regression analyses, BDI-II and neuropsychological scores were entered as predictors of TES diagnosis while also being used in making the TES diagnosis, which may have inflated findings of these specific predictors in these models. Nonetheless, our only consistent finding in this regard was the athlete's BDI-II score, and this variable remained significant after removing BDI-II as a diagnostic criterion. As stated earlier, our finding that depressive symptoms predict a TES diagnosis are not surprising in the context of the high base rate of depressive symptoms and the high frequency of depressive symptom criterion in the published TES criteria. Fifth, the Montenigro criteria attempt to increase the specificity of TES criteria to CTE using biomarker data, which was lacking in the current study. Lastly, although not the primary aim of the study, it should be noted that we are unable to provide an accurate estimate of the specificity and sensitivity of the TES criteria, as this would require *in vivo* biomarkers (which have not been validated) or autopsy-verification of CTE among both clinical and healthy control samples in addition to individuals with repetitive head-injury exposure. The only published data using autopsy-verification has not been peer reviewed (12). Still, we feel our findings underscore the potential for false-positive diagnoses of TES and CTE in those who may not be suffering from a neurodegenerative disease, and adds to the existing literature in this regard (13, 14).

Despite these limitations, the current study is the first to evaluate the frequency of TES diagnoses and TES criteria obtained through direct clinical evaluations of retired professional contact sport athletes, and the first to examine potential predictors of TES diagnosis. Our findings highlight the lack of specificity of the assumed features of CTE and the ease at which a retired player can achieve a diagnosis of TES, despite being clinically diagnosed as normal. We did not find TES diagnoses to be associated with greater head-injury exposure, and only depressive symptoms increased likelihood of a diagnosis, which is not surprising given the strong emphasis placed on potential psychiatric symptoms in the TES criteria. Our findings underscore the need for future study investigating the long-term cognitive correlates of head-injury exposure, the development of biomarkers to aid *in vivo* clinical diagnoses of CTE, and longitudinal clinicopathological studies of CTE that would add to our understanding regarding the potential relationship between clinical symptoms and CTE pathology.

## Data Availability Statement

The datasets presented in this article are not readily available because due to the nature of this research, participants of this study did not agree for their data to be shared publicly, so supporting data is not available. Requests to access the datasets should be directed to Munro Cullum, at munro.cullum@utsouthwestern.edu.

## Ethics Statement

The studies involving human participants were reviewed and approved by University of Texas Southwestern Medical Center Institutional Review Board and The University of Texas at Dallas Institutional Review Board. The patients/participants provided their written informed consent to participate in this study.

## Author Contributions

JS contributed to the study design, conceptualization, data collection, data analysis, and writing of the manuscript. ND, CL, JH, and CMC contributed to the study design, conceptualization, data collection, and writing of the manuscript. HR and LL contributed to the study design, interpretation of the results, and writing of the manuscript. All authors contributed to the article and approved the submitted version.

## Conflict of Interest

The authors declare that the research was conducted in the absence of any commercial or financial relationships that could be construed as a potential conflict of interest.
